# Genotype of Null Polymorphisms in Genes GSTM1, GSTT1, CYP1A1, and CYP1A1*2A (rs4646903 T>C)/CYP1A1*2C (rs1048943 A>G) in Patients with Larynx Cancer in Southeast Spain

**DOI:** 10.3390/cancers12092478

**Published:** 2020-09-01

**Authors:** Mariano Sánchez-Siles, Juan Pablo Pelegrín-Hernández, Diego Hellin-Meseguer, Yolanda Guerrero-Sánchez, Andrés Corno-Caparrós, Juan Cabezas-Herrera, Francisco Pastor-Quirante, Juan Alberto Fernández-Ruiz, Alfonso Aliaga-Sánchez, Mayra Lucero-Berdugo, Fabio Camacho-Alonso

**Affiliations:** 1 In Private Oral Surgery and Medicine Practice, 30001 Murcia, Spain; marianosasi@yahoo.es; 2 Otorhinolaryngology, University Hospital Virgen de la Arrixaca, 30005 Murcia, Spain; juanpablo.pelegrin@um.es (J.P.P.-H.); diegohellin.hellin@gmail.com (D.H.-M.); 3 Department of Human Anatomy and Psychobiology, University of Murcia, 30100 Murcia, Spain; 4 In Private Genetics Practice, ANCOR Laboratories, 03003 Alicante, Spain; aacorno@hotmail.com; 5 Molecular Therapy and Biomarkers Research Group, Clinical Analysis Service, IMIB, 30005 Murcia, Spain; juan.cabezas@carm.es; 6 Department of Anatomical Pathology, University of Murcia, 30100 Murcia, Spain; fpastor@um.es; 7 In Private Oral Surgery and Medical Practice, 07800 Ibiza, Spain; administracion@clinicafernandez.es; 8 Oral and Maxillofacial Surgery, University Hospital Reina Sofia, 30003 Murcia, Spain; info@clinicadraliaga.es; 9 Department of Oral Medicine, Catholic University of Murcia, 30107 Murcia, Spain; mayraluceroberdugo@gmail.com; 10 Department of Oral Surgery, University of Murcia, 30100 Murcia, Spain; fcamacho@um.es

**Keywords:** larynx cancer, single nucleotide polymorphisms, GSTM1, GSTT1, CYP1A1, CYP1A1–2

## Abstract

**Simple Summary:**

Epidemiological studies have shown that individual susceptibility to cancer is mediated by genetic and environmental factors. The aim of the present study is to evaluate the individuals’ metabolic genetic susceptibility to toxic habits (smoking and alcohol consumption) by detecting polymorphisms CYP1A1 rs1048943 T>C and CYPA1A2 rs4646903 A>G, and null polymorphisms in GSTM1 and GSTT1 genotypes, comparing a group of healthy control subjects with a population of larynx cancer patients from southeastern Spain. As results patients with larynx cancer present more gene GSTM1 and GSTT1 null polymorphisms, and CYP1A1 rs4646903 T>C polymorphisms.

**Abstract:**

Background: some types of cancer have been associated with the presence of single nucleotide polymorphisms (SNPs) of some genes that encode enzymes: glutathione-S transferase (GST), whose alteration leads to loss of function and a lower capacity to eliminate toxic GSTM1 and GSTT1 null genotypes; SNPs causing loss of function of CYP1A1 or CYP1A1–2 cytochrome P450 enzymes related with a lower capacity to deactivate hydrocarbons related to smoking, which involves a higher risk of developing some smoking-dependent cancers including larynx cancer. Objective: to compare the presence of null SNPs in genes GSTM1, GSTT1, and CYP1A1 rs 4646903 T>C, and CYP1A1–2 RS1048943 A>G in patients with hypopharyngeal and larynx cancer with a healthy control group. Materials and method: The study included a total of 80 patients with hypopharyngeal and laryngeal cancer and 23 healthy subjects. Genomic DNA was obtained from saliva samples, determining genotype GSTM1 (present +, or null −), GSTT1 (present + or null −). Polymorphisms (SNP) in CYP1A1 T>C (present + CC, or absent − TC/TT), and CYP1A1–2 A>G (present + GG, or absent − AG/AA). Results: the mean age of patients with larynx cancer was 62 years and of control subjects 63 years. Of the total sample, over 95% were men, and over 90% were smokers. The presence of null genotypes for GTM1 was 50% in patients with larynx cancer (*p* = 0.042), while GSTT1 was 88.75% (*p* = 0.002). CYP1A1 rs4646903 T>C polymorphisms were detected in 100% of cases of larynx cancer and 17.39% of healthy subjects (*p* > 0.001). Conclusions: patients with larynx cancer present more gene GSTM1 and GSTT1 null polymorphisms, and CYP1A1 rs4646903 T>C polymorphisms.

## 1. Introduction

Cancer continues to be one of the main causes of morbidity and mortality in the world, with approximately 18.1 million new cases in the world in 2018 [1]. Population estimates indicate that the number of new cases will increase in the next two decades to 29.5 million a year in 2040.

Epidermoid cancer of the head and neck is defined as a group of malign tumors that originate in the mucosa of the upper aerodigestive tract, representing 90% of all cancers of the head and neck. These are classified as: (a) oral cavity, pharynx, larynx; (b) nasal fossa/naso-sinusal cavity; (c) nasopharynx [2].

Oral squamous cancer is the most common malignant tumor of the head and neck and HPV-positive oropharynx cancer is increasing.

Around 3211 new cases of laryngeal cancer are expected in Spain, of which 2825 will be in men and the rest in women (data obtained from the SEOM report).

In the US, around 12,370 new cases of laryngeal cancer will be diagnosed (9820 men and 2550 women). Approximately 3750 people (3000 men and 750 women) will die from this disease (data obtained from the American Cancer Society) [3,4].

According to the data provided by GLOBALCAN [1], the continent with the highest incidence of laryngeal cancer is Asia, followed by Europe, Latin America, US, Africa and Oceania.

There is some racial disparity in incidence; for example, Afro-Americans usually develop this cancer at a younger age, presenting a higher incidence and higher rate of mortality in comparison with Caucasians [5,6]. Approximately 60% of patients have reached an advanced stage (stage III or IV) by the time of diagnosis [7]. Unfortunately, larynx cancer is one of the few oncological diseases in which the survival rate after 5 years has decreased during the last 40 years from 66% to 63%, although its general incidence is declining [2]. This highlights the need for research and innovation in this field.

Various risk factors are involved in the pathogenesis of larynx cancer. The most important are smoking and alcohol consumption. It has been shown that smoking presents a linear association with developing larynx cancer, with a 10–15 times higher risk among smokers than nonsmokers. Among smokers, higher numbers of cigarettes per day can increase the risk by up to 30 times [7,8].

Research has also demonstrated a relationship between alcohol consumption and the risk of larynx cancer [9]. Moreover, drinking and smoking together have a multiplying effect on the risk of developing larynx cancer [10]. It is believed that exposure to various environmental factors also increases the risk of larynx epidermoid carcinoma, including asbestos, aromatic polycyclic hydrocarbons, and textile dust [11,12]. Dietary factors have also been observed: red meat increases the risk of larynx cancer, while a varied diet of green vegetables and fruit has a potentially protective effect [13,14]. The role that gastroesophageal and laryngopharyngeal reflux may play in the disease is a controversial subject that remains under investigation [15,16].

Some research has demonstrated the effect of ALDH2 polymorphism on cancer risk in the Asian population. It is known that a metabolite of alcohol, acetaldehyde, interferes in DNA synthesis and repair. ALDH2 is an enzyme necessary for the elimination of this metabolite, so this polymorphism could contribute to the development of carcinogenesis [17].

Human papillomavirus (HPV) could be involved in the etiopathogenesis of oropharyngeal cancer. Initially, it was thought that HPV did not play any part in larynx cancer but numbers of studies have demonstrated the presence of HPV and/or p16 marker (cyclin-dependent kinase inhibitor 2A/multiple tumor suppressor) in laryngeal tumors, although the biological and prognostic significance of this finding remains unclear. It is estimated that the prevalence of HPV varies between 20% and 30% in larynx cancer, although this percentage varies widely between studies and depends on the detection method employed [18,19]. More research is needed to determine the clinical relevance of HPV/p16 in larynx cancer, which remains a controversial topic [20,21,22].

Although many people are exposed to the risk factors listed above, smoking and alcohol consumption are present in more than 80% of patients [8]. Larynx cancer only develops in a small number of persons exposed, which suggests the possibility of individual susceptibility [23,24]. It is presumed that susceptibility to larynx cancer is determined by individual differences in genetic factors contributing to procarcinogenic mechanisms. For this reason, it is only logical to suppose that genetic defects in the enzymes that process carcinogens may play some role in determining individual susceptibility to larynx cancer [25].

Most chemical carcinogens undergo metabolic activation in cells to form reagents that then interact with deoxyribonucleic acid (DNA) [26,27]. The metabolism of xenobiotic compounds acts in the cells by means of a wide spectrum of oxidative enzymes (phase I metabolism), which introduce polar groups (for example, hydroxyl groups) and make the molecules adequate substrates for conjugation (phase II metabolism). The resulting conjugate is substantially more water-soluble than the original compound and so more easily secreted [28,29].

Single nucleotide polymorphisms (SNP) are considered to be variations linked to a single nucleotide within DNA and may be found in more than one region of a gene sequence. SNP usually affects two alleles and is considered one of the most predominant variations in the human genome and is an invaluable source of information in biomedical research [30,31]. At the same time, SNPs offer great potential as both diagnostic tools and therapeutic biomarkers [32,33].

Genetic polymorphisms of a single nucleotide (SNP) have been investigated as a means of identifying individual susceptibility to many types of cancer [33,34] including larynx carcinoma [35]. Although most chemical carcinomas are inactive, they can become bioactive through phase I CYP. For example, benzopyrene can be metabolized and transformed into mutagenic benzoyl peroxide [36,37]. CYPs are the principal enzymes in cytochrome P45O, which metabolizes polycyclic aromatic hydrocarbons [38]. Among all the research into CYPs, the most widely investigated in terms of carcinogenic xenobiotics have been the SNPs of CYP1A1 rs1048943 A>G and CYP1A1 rs4646903 T>C [39]. SNP of CYP1A1 rs1048943 A>G involves isoleucine to valine amino acid substitution at codon 462, leading to CYP1A1 exon 7 polymorphism. CYP1A1 rs4646903 T>C is characterized by thymine mutation into cytosine in nucleotide 3801 in the gene’s 3′ region. These genetic changes play an important role in xenobiotic metabolism and in an individual’s susceptibility to many types of cancer [40,41].

Glutathione S-transferases (GST) are a family of phase II enzymes, which, through conjugation with glutathione, metabolize carcinogenic compounds that are found in tobacco smoke, products of hydrocarbon combustion, and diet [42,43]. These enzymes are encoded by different gene loci, which are known to present polymorphisms that can compromise their functionality. These enzymes also provide protection against oxidative stress, as well as xenobiotic phase II metabolism. GSTM1 and GSTT1 genes belonging to GSTs have been studied extensively due to their important detoxification function and high-frequency polymorphisms [44].

GSTM1 and GSTT1 homozygous deletions are associated with reduced function of xenobiotics detoxification, increased susceptibility to cytogenetic damage, and increased risk of cancer [45].

GSTM1 and GSTT1 null genotype results in incapacity to detoxify carcinogens related to tobacco smoke, which can increase the risk of smoking-related cancer, such as lung, bladder cancer and head and neck cancer [45,46].

GSTM1and GSTT1genotypes play an important role in an individual’s susceptibility to larynx cancer, as GST enzymes are detected site-specifically in the head and neck mucosa; the larynx mucosa expresses the highest concentration of GST isoform [45,47].

Previous studies have shown that a homozygous deletion or null genotype of the GSTM1 and GSTT1 gene that results in loss of the enzyme’s function and so increases susceptibility to head and neck cancer [45,47,48].

The aim of this study was to determine the presence of SNPs responsible for the loss of function of GSST1, GSTM1, CYP1A1 and CYP1A2 genes, as markers of risk, in a group of smokers and drinkers with larynx cancer.

## 2. Materials and Method

### 2.1. Recruitment and Patient Characteristics

The study protocol was approved by the University of Murcia Ethics Committee (1837/2018) and carried out between July 2017 and October 2019. This transversal retrospective case-control study had a study population of patients with clinical and anatomopathological diagnosis of laryngeal epidermoid carcinoma; all attended the University Hospital Virgen de la Arrixaca, Murcia, Spain.

Inclusion criteria were as follows: patients older than 18 years; patients resident in Healthcare Area 1 (West Murcia, Spain) or other nearby healthcare areas; anatomopathological diagnosis of laryngeal epidermoid carcinoma. All patients gave their informed consent to take part in the study. Smoking habit, alcohol habit or both.

Exclusion criteria were: patients aged younger than 18 years; patients of African or Asian (there are differences between ethnicities in the presence of polymorphisms) ethnicity; patients presenting primary tumor at a location other than the larynx, patients presenting benign tumors or nonepidermoid malignant tumors in anatopathological diagnosis (adenocarcinoma, papillary carcinoma, mucoepidermoid carcinoma, anaplastic carcinoma, melanoma, lymphoepithelioma and paraganglioma); patients unwilling to give their informed consent to take part.

Saliva samples were collected from the patients and control by the Hospital Otorhinolaryngology Service.

None of patients who fulfilled the inclusion criteria and were invited to take part in this study refused to do so. To calculate a representative sample size, a power of 80% was required (5% alpha level), using a Cohen’s d value of 0.30 for the effect site intended. A total of 80 consecutive patients with larynx/pharynx cancer were included in this transversal retrospective case control study, compared with a group of 23 healthy subjects without head and neck mucosal dysplasia or tumor diseases, or other tobacco-related diseases, but smoking habit, formed the control group.

### 2.2. Laboratory Analysis

The analytic procedure consisted of two phases; the first was as follows: A, homogenized, DNase-free DNA in sterile distilled water was obtained from saliva samples from both cases and control, and conserved until use in a test tube containing ethylenediaminetetraacetic acid (EDTA) at 4 °C. In the second phase, B, polymerase chain reaction (PCR) amplification was performed with oligonucleotides of the regions containing the SNPs to be analyzed and then the resulting amplicon was digested with restriction enzymes PCR-RFLP.

A. DNA extraction was performed by a method that consisted of a digestion process with 2 mL proteinase K (20 mg/mL) (Thermo Fisher Scientific, Vilnius, Lithuania) at 55 °C for 2 h with 200 μL digestion buffer in a TS-100 Biosan thermoshaker. The samples were centrifuged at 12,000 rpm (15,300 g) for 5 min removing the supernatant and adding 1 mL of 70% cold alcohol; after vortexing, it was centrifuged again at 12,000 rpm for 1 min, removing the supernatant. The precipitated DNA was left to dry at room temperature and finally resuspended in 60 μL sterile distilled water for Braun injectables and incubated at 40 °C for 1 h. The samples were quantified determining their purity with a Biophometer 631 spectrophotometer (Eppendorf, Hamburg, Germany).

B. Genotyping of polymorphisms: null in GSTM1, GSTT1, CYP1A1*2A (rs4646903;3798 T>C, T6235C, MspI), CYP1A1*2C (rs1048943; Ile462Val; g.2454 A>G) and CYP1A1. To genotype SNPs in CYP1A1 and GSTT1 and GSTM1, oligonucleotides were designed with Primer3Web version 4.0.0 software. A master mix was prepared (Buffer amplification, a mixture of ATP, CTP, GTP, TPP, Cl_2_Mg 5 mM, and nucleotides) in a 25 μL reaction volume amplified in a SimpliAmp thermocycler (Life Technologies, Carlsbad, CA, USA) with 1 U/μL Taq polymerase (Biotools Labs. SA, Madrid, Spain) using the following PCR sequence: 5 cycles at 94 °C for 10 min. (94 °C—30 s, 60 °C—30 s, 72 °C—45 s); 20 cycles (94 °C—30 s, 60 °C [0.5 °C/cycle]—30 s, 72 °C—45 s); and 10 cycles (94 °C—30 s, 50 °C—30 s, 72 °C—45 s); with samples left for 10 min at 72 °C at the end. Each series of samples was amplified with negative control amplification using water as a template and positive control of different genotypes for each SNP analyzed. For null SNPs of GSTM1 and GSTT1, amplifications of 215 and 150 bp, respectively, were produced in positive cases but no amplification was performed in negative cases. For CYP1A1 alleles *2A rs 46469 03, the enzyme Msp I generated a homozygous TT amplicon of 150 bp, and a cut of 119 + 31 bp in homozygous CC. For CYP1A1 alleles *2C rs 1048943, the restriction enzyme BsrDI at 55 °C generated an amplicon of 124 + 84 bp in homozygous wild-type AA and an uncut pattern of 208 bp in mutant. The samples were passed through agarose gels at 2%. The results were read under ultraviolet light using an UVI-Tec Transilluminator with software (UVI-DOC V.97, UVItec, Rugby, UK) for registration and analysis of the pattern and size of the fragments obtained. The results were replicated in a random 10% of the data.

### 2.3. Statistical Analysis

Data were analyzed using the SPSS version 20.0 statistical package (SPSS Inc., Chicago, IL, USA). A descriptive study was made of each variable. The associations between different qualitative variables were studied using Pearson’s chi-squared test. The Student t-test for two independent samples was applied to quantitative variables, in each case determining whether the variances were homogeneous. Adjusted odds ratios and confidence intervals were calculated using multiple logistic regression models considering the binaries “*GSTM1* present/absent,” “*GSTT1* present/absent,” “*CYP1A1* present/absent”, and “*CYP1A1–2* present/absent” as outcome variables; and using age, smoking, alcohol, and presence of larynx/pharynx cancer. Statistical significance was accepted for *p* ≤ 0.05.

## 3. Results

Of the 80 patients with larynx cancer, 75 had glottic and superglottic larynx cancer and five cases were diagnosed with hypopharyngeal cancer. The mean ages in case and control (*n* = 23) groups were 62.55 ± 9.86 and 63.87 ± 7.94, respectively. Almost all the patients with cancer (97.50%) were men, while 95.65% of control subjects were men. Both groups were homogeneous in terms of age, sex, smoking, and alcohol consumption (Table 1).

The most frequent location of laryngeal tumors was the glottic 47/75 (62.67%). As for laryngeal tumor size, 26/75 (34.76%) were T3 and 15/75 (20%) T4. Table 2 shows clinical characteristics including ganglion affectation and TNM staging. Regarding polymorphism detection in GSTM1 genotypes, patients presenting GSTM1 did not show deletion (null) polymorphism, whereby the gene maintains its detoxification function for potentially toxic compounds. This was observed in 73.91% of control subjects and in 50% of cancer patients (*p* = 0.042).

Null GSST1 genotype was present in 88% of cancer patients, so the GSTT1 gene was not present, resulting in no enzyme detoxification function (*p* = 0.002) (Table 3).

Table 4 represents a logistic regression model and confidence intervals, showing how smoking present a statistically significant relationship with presence or absence of the GSMT1 genotype (*p* = 0.008). Alcohol consumption and the presence of tumors were also found to show a relation with the absence of the GSST1 genotype (*p* = 0.023 and *p* = 0.003, respectively)

Regarding the existence of SNPs in genes that encode CYP1A1 and CYP1A2 enzymes of cytochrome P450, for polymorphisms CYP1A1 rs4646903 T>C, the TT or CT wild-type without mutation was detected in 82.61% of healthy control subjects (*p* < 0.001). CC polymorphism was present in 100% of patients with larynx cancer with statistically significant difference (*p* < 0.001).

As for CYP1A1 rs1048943 A>G polymorphism, for CYPA1A2 genotype, GG polymorphisms were detected in 80% of larynx cancer cases although without statistically significant difference (*p* = 0.075) (Table 5).

Table 6 represents a logistic regression model of the presence or absence of polymorphism of a single nucleotide in CYP1A1 and CYPA1–2 genes. The presence of a tumor was found to be the variable showing the strongest (statistically significant) relation with the presence of these SNPs (*p* < 0.001).

## 4. Discussion

The aim of the present study was to evaluate the individuals’ metabolic genetic susceptibility to toxic habits (smoking and alcohol consumption) by detecting polymorphisms CYP1A1 rs1048943 T>C and CYPA1A2 rs4646903 A>G, and null polymorphisms in GSTM1 and GSTT1 genotypes, comparing a group of healthy control subjects with a population of larynx cancer patients from southeastern Spain.

Epidemiological studies have shown that individual susceptibility to cancer is mediated by genetic and environmental factors [47,48]. It is known that exposure to carcinogens is an important risk for developing cancer. Various studies have demonstrated that smoking and drinking alcohol have a strong influence on laryngeal carcinoma. Some 80% of larynx cancer patients have antecedents of smoking and alcohol abuse, and the risk of cancer is proportional to the duration and quantity of smoking and drinking [48]. In the present study, more than 90% of cases and control subjects were smokers and over 60% drank alcohol [49].

The present work proposed the hypothesis that metabolism modulation by carcinogenic chemical agents, whether through activation or detoxification, are largely under genetic control. This would constitute a mechanism that determines individual susceptibility. Three principal families of proteins exhibit glutathione transferase activity: cytosolic GST, mitochondrial GST, and microsomal GST. Cytosolic GSTs suffer polymorphisms in humans and could be the main cause of interindividual variations in responses to xenobiotics [40].

Null mutations of GSTM1 and GSTT1 phase II enzymes have been shown to halt enzyme activity resulting in greater susceptibility to environmental toxins and carcinogens related to an increased risk of cancer [40,49].

The literature includes several studies that have shown that null polymorphisms in GSTM1 could increase the risk of cancer of the head and neck, as well as oral cancer [45,47], breast cancer [50], and lung cancer [51,52]. However, various meta-analyses have not detected any marked association between null GSTM1 mutation, and hepatocellular carcinoma [53] esophageal cancer [54] or prostate cancer [54]. The findings for larynx cancer have shown that GSTM1 null polymorphism cancels the enzyme’s function, which is associated with the development of larynx cancer. The present results are similar to those of other authors who have also demonstrated an increased risk of larynx cancer in individuals with null GSTM1 genotype [55,56].

Most studies and meta-analyses consider that null GSTT1 polymorphisms are not associated with increased risk of larynx cancer [57] and oral cancer [58,59]. One investigation obtained results pointing to greater susceptibility among smokers and drinkers, associated with the absence of GSST1 enzyme in an Asian population [60]. The present study found that most cancer cases and control subjects presented null GSST1 genotype polymorphism: 14/23 (60.86%) and 71/80 individuals (88.75%), respectively. Although statistically significant differences were detected (*p* = 0.002), these could be due to the difference in sample sizes between healthy and control subjects as there is no relation between larynx cancer and null GSST1 polymorphism. Meanwhile, associations have been demonstrated between null GSTT1 genotype and lung cancer [51], brain tumor [60] colorectal cancer [61] gastric cancer [62], leukemia [63] and cancer of the head and neck [43].

CYP1A1 is a gene that encodes the cytochrome enzyme. Its main function is to metabolize aromatic hydroxylase hydrocarbon. CYP1A1 is one of the most important isoenzymes in the CYP450 enzyme system. Human CYP1A1 can catalyze human polycyclic aromatic hydrocarbons, active carcinogens, and other exogenous compounds [37].

In the present study, all 80 (100%) of the cases of larynx cancer presented rs4646903 CYP1A1 T>C polymorphism (*p* > 0.001). All cases were CC homozygous. There were no cases of heterozygosity (TC) and none of wild-type heterozygosity (TT). Among the healthy control subjects, 82.61% were TC/CC, with statistically significant difference (*p* < 0.001).

A study by Zhu Li et al. investigated (SNP) polymorphisms for the genes XPG, CYP1A1, OGG1, ERCC5, ERCC1, MMP2 and MMP9 in a group of 200 patients with larynx cancer associated with heavy smoking in Shanghai (China), finding an association between SNP of genes CYP1A1 and MMP9 and larynx cancer. The authors proposed that these SNPs act as markers of an increased susceptibility to developing larynx cancer among smokers [64].

Zhen et al. (2016) performed a meta-analysis of ten clinical trials including a total of 748 Asian patients with larynx cancer and 1558 control subjects. They found that subjects presenting SNPs for CYP1A1 rs1048943 and rs4646903 had an increased risk of larynx cancer [59].

In addition to lung cancer, colorectal cancer, breast cancer, leukemia, esophageal carcinoma and prostate cancer [65,66], CYP1A1 polymorphisms can also lead to other diseases such as ulcerative colitis, colorectal adenoma, myocardial infarction, etc. [66,67].

In the case of CYP1A1–2 rs1048943 A>G, no statistically significant differences were found between groups (*p* = 0.075); both control subjects and larynx cancer patients presented high rates of SNP homozygosity for GG genotype: 22 (95.65%) control subjects and 64 (80%) cancer patients, respectively. Regarding CYPA1–2 rs1048943 A>G SNP, several previous studies have reported a significant increase in the risk of larynx cancer among individuals carrying GG alleles in an Asian population, but not among Caucasians presenting GG polymorphism compared to those presenting wild-type homozygous AA (without mutation). The same polymorphism would appear to play various roles in CYP1A in terms of susceptibility to cancer among different ethnicities, although SNP frequencies differ between Caucasians and Asians [59]. These findings may be extrapolated to the present investigation, in which a high frequency of homozygous GG genotype was observed in both the control group and among laryngeal and hypopharyngeal cancer patients, as 22 (95.65%) control subjects and 64 (80%) cases of larynx cancer presented genotype GG (*p* = 0.075).

## 5. Conclusions

In spite of this study’s limitations and the need for prospective studies with larger sample sizes, the findings suggest that the presence of GSTM1 and GSTT1 null polymorphisms and CYP1A1 3 rs4646903 T>C are associated with a higher risk of developing larynx cancer in male Caucasian smokers in southeastern Spain.

## Figures and Tables

**Table 1:** Study groups’ demographic characteristics and smoking and alcohol habits (Student t-test and Pearson χ^2^).

Characteristics	Larynx/Hypopharynx Cancer (*n* = 80)	Healthy Controls (*n* = 23)	OR (95% CI)	*p*-Value
Age: mean ± SD *	62.55 ± 9.86	63.87 ± 7.94	2.24 (5.76–3.12)	0.557
Sex: *n* (%)			1.77 (0.15–20–47)	0.642
Male	78 (97.50)	22 (95.65)		
Female	2 (2.50)	1 (4.35)		
Smoking behavior: *n* (%)			0.85 (0.16–4.53)	0.850
Yes	74 (92.50)	21 (91.31)		
No	6 (7.50)	2 (8.69)		
Alcohol consumption: *n* (%)			1.12 (0.42–2.96)	0.812
Yes	50 (62.50)	15 (65.21)		
No	30 (37.50)	8 (34.79)		

* SD = standard deviation.

**Table 2:** Baseline characteristics of larynx/pharynx cancer patients.

Characteristics	Larynx Cancer (*n* = 75) *n* (%)	Pharynx Cancer (*n* = 5) *n* (%)
Tumour site		
Supraglottic	28 (37.33)	-
Glottic	47 (62.67)	-
Hypopharynx	-	5 (100.00)
T Stage		
1	14 (18.66)	0 (0)
2	20 (26.67)	0 (0)
3	26 (34.67)	2 (40.00)
4	15 (20.00)	3 (60.00)
N Stage lymph nodes		
0	54 (72.00)	0 (0)
1	8 (10.67)	0 (0)
2	11 (14.67)	1 (20.00)
3	2 (2.66)	4 (80.00)
Clinical Stage		
I	14 (18.66)	0 (0)
II	20 (26.67)	0 (0)
III	16 (21.34)	1 (20.00)
IV	25 (33.33)	4 (80.00)

**Table 3:** Distribution of genotypes of *GSTM1* and *GSTT1* among healthy controls and patients with larynx or hypopharynx cancer (Pearson χ^2^ test).

Genotype	Healthy Controls (*n* = 23) *n* (%)	Larynx/Hypopharynx (80) *n* (%)	OR (95% CI)	*p*-Value	Larynx Cancer (*n* = 75) *n* (%)	OR (85% CI)	*p*-Value	Hypopharynx Cancer (*n* = 5) *n* (%)	OR (95% CI)	*p*-Value
*GSTM1*			2.83 (1.01–7.92)	0.042		2.75 (0.98–7.76)	0.049		4.25 (0.56–31.93)	0.141
Absence or null	6 (26.09)	40 (50.00)			37 (49.33)			3 (60.00)		
Presence	17 (73.91)	40 (50.00)			38 (50.67)			2 (40.00)		
*GSTT1*			5.07 (1.71–15.04)	0.002		4.71 (1.58–14.01)	0.003		1.64 (1.18–2.28)	0.090
Absence or null	14 (60.86)	71 (88.75)			66 (88.00)			5 (100.00)		
Presence	9 (39.14)	9 (11.25)			9 (12.00)			0 (0)		

**Table 4:** Logistic regression model for “*GSTM1* present/absent” and “*GSTT1* present/absent”.

Variables	*GSTM1* Present/Absent			*GSTT1* Present/Absent		
	Odds Ratio	95% Confidence Interval	*p*-Value	Odds Ratio	95% Confidence Interval	*p*-Value
Age (≤65/>65)	0.73	0.31–1.69	0.465	0.38	0.11–1.44	0.157
Smoking	1.93	1.59–2.35	0.008	0.61	0.11–3.28	0.563
Alcohol	0.84	0.37–1.91	0.690	5.87	1.27–27.18	0.023
Larynx/hypopharynx cancer	2.83	1.01–7.92	0.047	5.07	1.71–15.04	0.003

**Table 5:** Distribution of genotypes of *CYP1A1* and *CYP1A1–2* polymorphism among healthy controls and patients with larynx or pharynx cancer (Pearson χ^2^ test). 19 (82.61) 4 (17.39).

Genotype	Healthy Controls (*n* = 23) *n* (%)	Larynx/Hypopharynx (80) *n* (%)	OR (95% CI)	*p*-Value	Larynx Cancer (*n* = 75) *n* (%)	OR (85% CI)	*p*-Value	Hypopharynx Cancer (*n* = 5) *n* (%)	OR (95% CI)	*p*-Value
*CYP1A1* rs4646903			1.21 (1.01–1.46)	<0.001		1.21 (1.01–1.46)	<0.001		1.21 (1.01–1.46)	0.314
(+): CC	4 (17.39)	80 (100.00)			75 (100.00)			5 (100.00)		
(-): TC/TT	19 (82.61)	0 (0)			0 (0)			0 (0)		
*CYP1A1–2* rs1048943			0.18 (0.02–1.45)	0.075		0.16 (0.02–1.34)	0.060		1.04 (0.95–1.14)	0.635
(+): GG	22 (95.65)	64 (80.00)			59 (78.66)			5 (100)		
(-): AG/AA	1 (4.35)	16 (20.00)			16 (21.34)			0 (0)		

**Table 6:** Logistic regression model for “*CYP1A1* polymorphisms present/absent” and “*CYP1A1–2* polymorphisms present/absent”.

Variables	*CYP1A1* Present/Absent			*CYP1A1–2* Present/Absent		
	Odds Ratio	95% Confidence Interval	*p*-Value	Odds Ratio	95% Confidence Interval	*p*-Value
Age (≤65/>65)	0.73	0.07–7.31	0.790	0.91	0.29–2.84	0.872
Smoking	0.91	0.86–0.97	0.554	0.56	0.11–3.05	0.505
Alcohol	0.57	0.07–4.23	0.584	0.33	0.11–0.98	0.046
Larynx/hypopharynx cancer	0.19	0.12–0.28	<0.001	0.18	0.02–1.45	0.108
